# Risk factors for electroconvulsive therapy-induced fever: a retrospective case-control study

**DOI:** 10.3389/fpsyt.2024.1530533

**Published:** 2025-01-24

**Authors:** Can-Jin Deng, Jian-Wen Yang, Zi-Zhe Liu, Ting Ning, Sha Nie, Xiong Huang, Xin-Hu Yang, Xing-Bing Huang, Wei Zheng

**Affiliations:** ^1^ Psychiatry Department, The Affiliated Brain Hospital, Guangzhou Medical University, Guangzhou, China; ^2^ Key Laboratory of Neurogenetics and Channelopathies of Guangdong Province and the Ministry of Education of China, Guangzhou Medical University, Guangzhou, China

**Keywords:** electroconvulsive therapy-induced fever, electroconvulsive therapy, risk factors, prevalence, adverse effects

## Abstract

**Background:**

Electroconvulsive therapy (ECT)-induced fever can significantly affect patient experience, treatment adherence, and the course of treatment. However, little is known about the prevalence of ECT-induced fever and its associated risk factors in patients with major mental disorders (MMD).

**Methods:**

This retrospective, case-control study included 113 cases and 226 age-matched controls (1:2). The case group comprised patients who exhibited an axillary temperature of ≥37.5°C (99.5°F) at least once within 24 hours post-ECT. Patients diagnosed with MMD underwent ECT between January 1, 2021, and December 31, 2021, at a large psychiatric hospital in China. Demographic and clinical data were extracted from the electronic chart management system (ECMS) for both groups.

**Results:**

The prevalence of ECT-induced fever in patients with MMD was 6.8% [113/1,674, 95% confidence interval (CI): 5.6% to 8.0%], with a session-based prevalence of 1.1% (130/11,570, 95% CI: 0.9% to 1.3%). Multivariate logistic regression analysis identified paliperidone [odds ratios (OR)=2.5, 95% CI: 1.2 to 4.9] as a risk factor, while quetiapine (OR=0.4, 95% CI: 0.3 to 0.8) was found to be protective. No significant association between etomidate and ECT-induced fever was observed in univariate analysis (*p*>0.05).

**Conclusions:**

This study found a relatively low prevalence of ECT-induced fever. Paliperidone was identified as a risk factor, while quetiapine had a protective effect. Etomidate was not significantly associated with ECT-induced fever in patients with MMD.

## 1 Introduction

Major mental disorders (MMD) encompass a range of mental health disorders, including schizophrenia, bipolar disorder (BD), and major depressive disorder (MDD) (1), which account for a heavy burden of disease (2–4). Globally, approximately 1 billion individuals suffer from MMD, accounting for 13% of the global burden of disease (5–7). MMD is related to heightened economic strain, elevated mortality rates, heightened suicidal behavior, and diminished quality of life (8, 9). Although pharmacotherapy represents the dominant treatment for MMD, it is frequently insufficient for many patients (10). As a result, non-invasive neurostimulation techniques, such as repetitive transcranial magnetic stimulation (rTMS) (11, 12), transcranial direct current stimulation (tDCS) (13, 14), magnetic seizure therapy (MST) (10, 15), and electroconvulsive therapy (ECT) (16, 17), are frequently employed in clinical settings to enhance treatment outcomes.

ECT, which induces brief, generalized seizures through electrical currents under general anesthesia, is one of the oldest and most effective non-invasive neurostimulation techniques (18–20). ECT, which was first introduced in China during the early 1950s (21), is crucial for treating different MMD, including mood disorders (e.g., MDD and BD) and psychotic disorders (e.g., schizophrenia) (19, 22, 23). According to the study by Tang et al. (21), 150,000 ECT sessions are conducted each year in China. Compared to pharmacotherapy and psychotherapy, ECT offers several advantages, such as rapid symptom improvement in cases of severe depression, psychosis, and catatonia, and a reduction in rehospitalization and suicide rates (24–27). However, ECT is also associated with specific side effects, which can deter some patients from opting for this treatment (28, 29).

Common side effects of ECT include transient memory impairment, headaches, and muscle pain, but not post-ECT fever (28–31). Post-ECT fever can negatively impact the patient’s treatment experience (32, 33). Moreover, it may lead to a decline in treatment adherence, a fundamental factor in the clinical effectiveness of any intervention (32, 33). The onset of fever after ECT can delay the overall treatment process, hindering timely and effective management of MMD (34, 35).

The prevalence of ECT-induced fever in patients with MMD has been reported to vary significantly across studies (32, 33, 36, 37). For instance, Xiao et al. (36) conducted a retrospective study involving 76 patients with mental disorders, finding that 4 of 76 (5.3%) experienced ECT-induced fever. In contrast, Xie et al. (37) reported a much higher prevalence of 45.2% (56 of 124 patients diagnosed with schizophrenia or mood disorders) in their retrospective survey. The identification of risk factors for ECT-induced fever has been inconsistent across studies (33, 38). For instance, Jo et al. (33), in their retrospective chart review study involving 319 patients, found no significant difference in the rate of etomidate use between ECT sessions with fever and control sessions without fever (27.8% vs. 21.5%), indicating that etomidate was not a significant risk factor. However, a controlled study involving patients with MMD found that the prevalence of ECT-induced fever was significantly higher in the etomidate group (n=30) compared to the propofol group (n=30) (46.7% vs. 16.7%) (38), indicating that etomidate could be a significantly relevant factor.

Given the wide variation in reported prevalence rates and the conflicting evidence regarding associated risk factors, further research with larger sample sizes is necessary to clarify the prevalence of ECT-induced fever and identify potential risk factors in patients with MMD. This study aimed to 1) investigate the prevalence of ECT-induced fever, and 2) identify and compare potential risk factors associated with ECT-induced fever in patients with MMD.

## 2 Methods

### 2.1 Setting and participants

This single-center retrospective case-control study, part of a larger clinical project on ECT in psychiatry, was conducted at the Affiliated Brain Hospital, Guangzhou Medical University. This institution is an affiliated teaching hospital and a psychiatric center with 1,800 beds in Guangzhou, China. The Ethics Committee of the Affiliated Brain Hospital, Guangzhou Medical University, approved the study protocol (approval code: 2021001), with an exemption from informed consent due to the retrospective nature of the chart review.

The inclusion criteria for the case group were: 1) male or female inpatients diagnosed with schizophrenia, BD, or MDD as per the International Classification of Diseases, Tenth Revision (ICD-10); and 2) those who experienced ECT-induced fever [defined as an axillary temperature ≥37.5°C (99.5°F)] (39, 40) on at least one occasion within 24 hours after ECT. Patients were excluded if they had a pre-existing fever before the ECT session due to conditions such as infections [including bacterial, fungal, and coronavirus disease-2019 (COVID-19)], inflammatory diseases, or hematological disorders.

Patients who underwent ECT without fever [axillary temperature <37.5°C (99.5°F)] (39, 40) during the same hospitalization period were eligible for the control group. Control participants were matched by age (± 4 years) to the case group in a 2:1 ratio, following previous recommendations (41).

### 2.2 Data collection

Demographic information, clinical characteristics, and drug prescriptions for all discharged patients were collected in the hospital’s electronic chart management system (ECMS), which was established in January 2010. Data collection covered the case and control groups, focusing on demographic characteristics, clinical variables, and medications administered during ECT sessions. This study was conducted over one year between January 1, 2021, and December 31, 2021. Three trained researchers (C-JD, J-WY, and Z-ZL) were responsible for extracting data from the ECMS and compiling a database for analysis.

### 2.3 Prevalence of fever

Following a previous study (33), fever sessions were defined as ECT sessions in which the patient developed a fever within 24 hours after receiving ECT, while control sessions were referred to as ECT sessions without fever. The prevalences of fever sessions and fever following ECT were calculated by dividing the fever session counts and the number of patients with fever after ECT by the total number of ECT sessions and patients, respectively. In this study, we focused on examining the prevalence of ECT-induced fever and fever sessions. Thus, the prevalences of ECT-induced fever and fever sessions were determined by dividing the number of patients in the case group and their fever sessions by the total number of patients without pre-existing fever before ECT and their total ECT sessions, respectively.

### 2.4 ECT procedure and anesthesia

Before the first ECT session, all patients who are scheduled for ECT underwent a pre-ECT assessment, which included electroencephalography (EEG), chest x-ray, electrocardiogram (ECG), blood tests, urine analysis, psychiatric evaluation, and physical examination. Patients were required to fast and void for at least 8 hours before each ECT session. Moreover, a negative COVID-19 polymerase chain reaction (PCR) test was mandatory. ECT was administered using the MECTA spECTrum 5000Q device (Mecta Corporation, Tualatin, OR, USA) with bilateral electrode placement. The initial stimulus dose was determined using the half-age method (42, 43) and was adjusted throughout the treatment course.

Atropine (0.5 mg) was administered intravenously. As determined by the anesthetist’s clinical expertise, anesthesia was induced with either 1.5–2.0 mg/kg of propofol or 0.33–0.50 mg/kg of etomidate. Muscle relaxation was achieved using 0.8–1.0 mg/kg of intravenous succinylcholine. Vital signs, including blood pressure, oxygen saturation, and pulse, were monitored closely throughout the procedure.

### 2.5 Statistical analysis

Statistical analysis was conducted using Statistical Package for the Social Sciences (SPSS) (version 23.0, International Business Machines Corporation, New York, USA) for Windows. The Kolmogorov–Smirnov test was used to assess the normality of continuous data. Continuous variables are presented as mean and standard deviation (SD), while categorical data are expressed as frequencies and percentages (%). The univariate analysis compared the case and control groups’ potential risk factors for ECT-induced fever. The two-tailed Student’s *t*-test was applied for normally distributed continuous data, the Mann–Whitney U test for non-normally distributed continuous data, and chi-squared test for categorical data. Variables with a *p*-value of less than 0.05 in the univariate analysis were then included in a multivariate logistic regression analysis. The model’s validity was confirmed through the Omnibus (*p*<0.05) and Hosmer–Lemeshow (*p*>0.05) tests. The multivariate analysis results are presented with regression coefficient (B), standard error (SE), Wald statistic (Wald), degrees of freedom (df), significant level (Sig.), odds ratio (OR), and the 95% confidence interval (CI) of the OR. Statistical significance was defined as *p*<0.05 (two-tail test).

## 3 Results

### 3.1 Prevalence of fever

**Figure 1:**
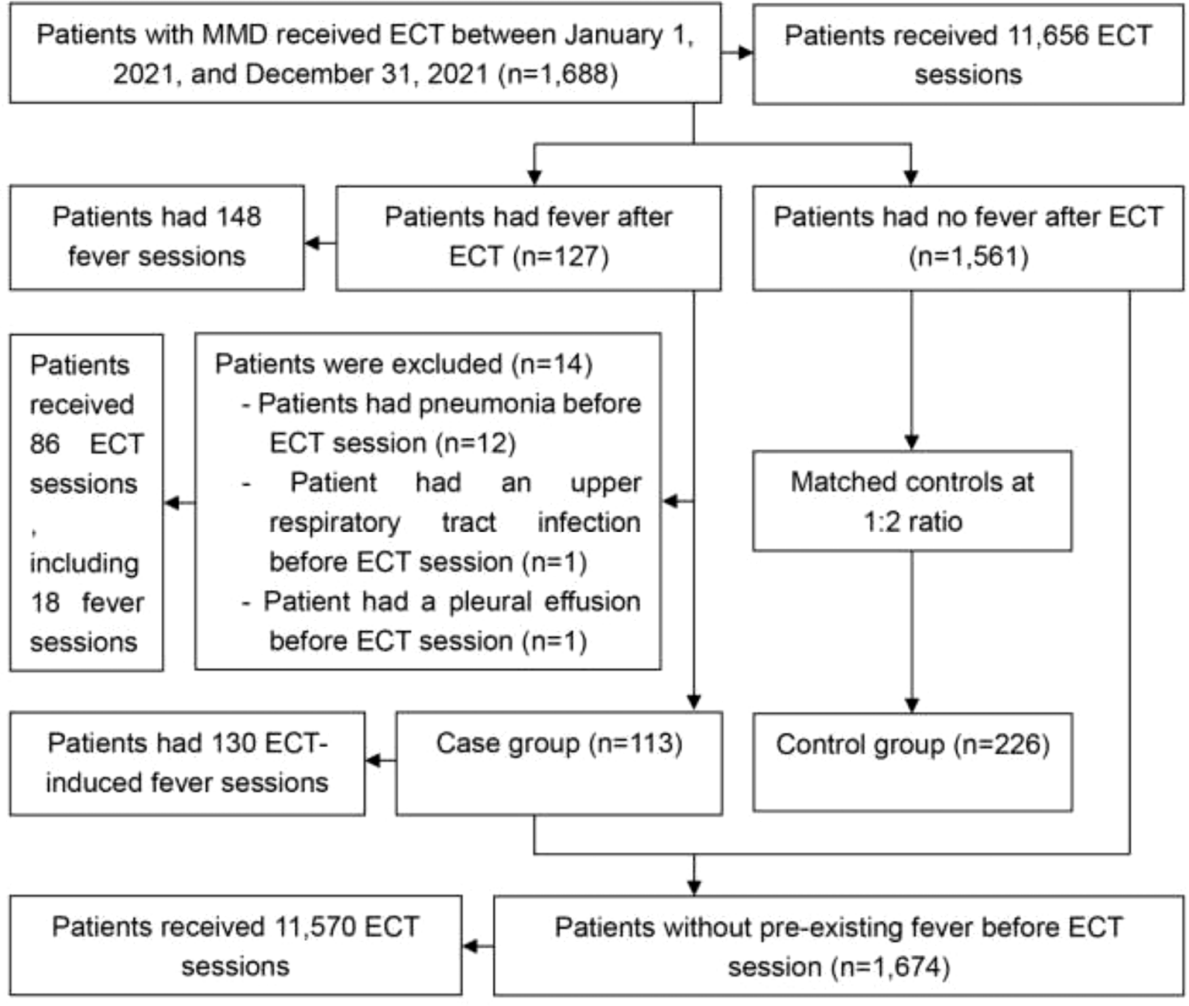
Study participant flow chart. ECT, electroconvulsive therapy; MMD, major mental disorders.

As illustrated in **Figure 1**, 1,688 inpatients with MMD underwent 11,656 ECT sessions. Among them, 127 patients experienced 148 fever sessions, resulting in a post-ECT fever prevalence of 7.5% (127/1,688, 95% CI: 6.2% to 8.8%) and a fever session prevalence of 1.3% (148/11,656, 95% CI: 1.1% to 1.5%). Of these, 14 patients (86 ECT sessions, including 18 fever sessions) had pre-existing fevers due to unrelated factors (**Figure 1**). After excluding these patients, the final case group comprised 113 patients with 130 ECT-induced fever sessions. Consequently, the prevalence of ECT-induced fever was 6.8% (113/1,674, 95% CI: 5.6% to 8.0%), and the prevalence of ECT-induced fever sessions was 1.1% (130/11,570, 95% CI: 0.9% to 1.3%).

### 3.2 Demographic and clinical characteristics of the study sample

The case group was successfully age-matched to 226 controls (± 4 years) in a 1:2 ratio. A comparison of demographic and clinical characteristics between the two groups is summarized in **Table 1**. Patients in the case group showed a substantially higher usage rate of paliperidone and a lower usage rate of quetiapine compared to the control group (both *p*=0.002). No considerable differences were observed between the groups in other demographic or clinical variables (all *p*>0.05).

**Table 1:** Demographic and clinical characteristics of the study sample.

Variables	Total Sample (N=339)		Case Group (N=113)		Control Group (N=226)		Statistics		
	n	%	n	%	n	%	χ^2^	df	*p*
Male	126	37.2	45	39.8	81	35.8	0.51	1	0.47
Diagnosis							0.23	2	0.89
MDD	111	32.7	37	32.7	74	32.7			
BD	143	42.2	46	40.7	97	42.9			
Schizophrenia	85	25.1	30	26.5	55	24.3			
Comorbidities									
Thyroid dysfunction	3	0.9	1	0.9	2	0.9	0	1	1.00
Diabetes	3	0.9	1	0.9	2	0.9	0	1	1.00
Hypertension	8	2.4	5	4.4	3	1.3	1.94	1	0.16
Administration of anesthetics							2.13	1	0.14
Etomidate	164	48.4	61	54.0	103	45.6			
Propofol	175	51.6	52	46.0	123	54.4			
Benzodiazepine									
Alprazolam	49	14.5	14	12.4	35	15.5	0.58	1	0.45
Lorazepam	94	27.7	27	23.9	67	29.6	1.24	1	0.27
Diazepam	46	13.6	14	12.4	32	14.2	0.20	1	0.65
Oxazepam	72	21.2	21	18.6	51	22.6	0.71	1	0.40
Clonazepam	15	4.4	5	4.4	10	4.4	0.00	1	1.00
Estazolam	6	1.8	1	0.9	5	2.2	0.19	1	0.66
Antipsychotic drugs									
Paliperidone	38	11.2	21	18.6	17	7.5	9.26	1	**0.002**
Quetiapine	94	27.7	19	16.8	75	33.2	10.08	1	**0.002**
Olanzapine	107	31.6	38	33.6	69	30.5	0.34	1	0.56
Clozapine	30	8.8	9	8.0	21	9.3	0.17	1	0.69
Antidepressant drugs^a^									
scitalopram	17	5.0	5	4.4	12	5.3	0.12	1	0.73
	Mean	SD	Mean	SD	Mean	SD	Z	df	*p*
Age (years)	26.9	12.1	26.6	12.6	27.1	11.9	-0.77	—^b^	0.44
Duration of illness (months)	66.7	72.3	60.0	76.1	70.0	70.3	-1.91	—^b^	0.06

Bold values indicate *p*<0.05.

a Given that only escitalopram was recorded and analyzed in previous studies, thus only escitalopram was collected in this study.

b Mann–Whitney U test.

BD, bipolar disorder; df, degree of freedom; MDD, major depressive disorder; SD, standard deviation.

### 3.3 Factors independently associated with ECT-induced fever

Paliperidone and quetiapine were further analyzed through multivariate logistic regression analysis. The logistic regression model demonstrated a good fit, as indicated by the Omnibus test (*p*=0.001) and the Hosmer–Lemeshow test (*p*=0.65). The analysis revealed that both medications were independent factors for ECT-induced fever (**Table 2**). Patients taking paliperidone before ECT had a 1.5-fold higher risk (OR: 2.5, 95% CI: 1.2 to 4.9) of developing fever than those not on paliperidone (*p*=0.01). Conversely, the risk of fever was significantly lower in patients on quetiapine, with an OR of 0.4 (95% CI: 0.3 to 0.8), indicating a reduced likelihood of fever by 60% (*p*=0.01).

**Table 2:** Factors independently associated with electroconvulsive therapy-induced fever.

Variables	B	SE	Wald	df	Sig.	OR	95% CI for OR	
							Lower	Upper
Paliperidone	0.9	0.4	6.5	1	**0.01**	2.5	1.2	4.9
Quetiapine	-0.8	0.3	7.8	1	**0.01**	0.4	0.3	0.8

Bold values indicate *p*<0.05.

B, beta regression coefficient; CI, confidence interval; df, degrees of freedom; OR, odds ratio; SE, standard error; Sig., significance level (*p*-value); Wald, Wald statistic.

## 4 Discussion

To the best of our knowledge, this study is the first to report the prevalence and risk factors for ECT-induced fever in Chinese patients with MMD, utilizing a relatively large sample size (n=339) compared to prior research (32, 33, 36, 37). Critical findings include (1) a prevalence of 6.8% for ECT-induced fever and 1.1% for ECT-induced fever sessions among patients with MMD; (2) a significant association between paliperidone and quetiapine use and ECT-induced fever; and (3) no observed correlation between the use of etomidate and ECT-induced fever incidence. However, the prevalence and risk factors for ECT-induced fever are poorly investigated in the past three years.

The prevalence of ECT-induced fever in this study (6.8% among patients with MMD) is similar to those in prior research (32, 33, 36). For example, Xiao et al. (36) conducted a retrospective study involving 76 patients with mental disorders, reporting a 5.3% (4/76) incidence of ECT-induced fever. Similarly, a randomized controlled trial of 120 patients with MMD found that 8.3% (10/120) developed a fever following ECT (32). However, a retrospective study reported a considerably higher prevalence (45.2%) in patients with schizophrenia (n=76) or mood disorders (n=48) after ECT (37), which contrasts with our study’s findings (6.8%) and those from other studies (ranging from 5.3% to 8.8%) (32, 33, 36). These discrepancies may be attributable to variations in methodology, fever definition, and sample size across studies (32, 33, 36, 37). In clinical practice, ECT is associated with several side effects beyond fever. Memory impairment following ECT is reported in 18.0% to 72.8% of patients with MMD (28, 29, 44, 45), while 1.4% to 48.1% and 19.5% to 30.0% of patients experience headache and muscle pain, respectively (28, 29, 44, 46), after ECT. Therefore, ECT-induced fever should be considered as significant as the other common side effects. The exact mechanism behind ECT-induced fever remains unclear. One hypothesis is that mask ventilation, which can create airway pressures as high as 60 mmHg, may lead to fever by causing aspiration pneumonia (47, 48). A novel intraoperative ventilatory technique, transnasal humidified rapid-insufflation ventilatory exchange (THRIVE), which generates airway pressures below 7.4 cmH_2_O, has been introduced for ECT procedures (49–51). However, the impact of the THRIVE technique on the incidence of ECT-induced fever is yet to be reported.

In our study, patients not taking quetiapine had a higher likelihood of developing ECT-induced fever compared to those who did, consistent with previous research (33). Jo et al. (33) conducted a retrospective chart review study on 319 patients who underwent 2,928 ECT sessions in South Korea and found that fever sessions involved a significantly lower mean dose of quetiapine than sessions without fever (64.3 mg/day vs. 117.0 mg/day). This finding suggests that quetiapine administration during ECT may serve as a protective factor against fever. The activation of 5-hydroxytryptamine 2 (5-HT_2_) receptor may result in body temperature increase (52). Quetiapine has significant antagonistic effects on serotonin in 5-HT_2_ receptors (53). Moreover, quetiapine has been shown to inhibit hypothalamic-pituitary-adrenal (HPA) system activity in healthy subjects (54), which may prevent its overactivation and consequently decrease fever.

Regarding paliperidone, Jo et al. (33) reported similar mean dosages of paliperidone between ECT sessions with and without fever (9.50 mg/day vs. 7.46 mg/day), indicating no clear link between paliperidone use and fever development. However, in this study, the case group of patients with ECT-induced fever exhibited a substantially higher rate of paliperidone administration during the ECT procedure than the control group without fever (18.6% vs. 7.5%). Moreover, multivariate logistic regression analysis indicated a positive association between paliperidone administration and ECT-induced fever (OR=2.5). These conflicting findings suggested that the occurrence of ECT-induced fever could be related to the dosage of paliperidone, which was not collected in this study. The biological mechanisms by which paliperidone leads to ECT-induced fever have not been sufficiently investigated. The effect of paliperidone in reducing dopamine levels in the brain may disrupt the normal thermoregulatory balance, increasing susceptibility to fever during ECT (55–57). Moreover, paliperidone may cause fever by interacting with the immune system to produce an excessive inflammatory response (58).

Our analysis did not reveal any significant association between etomidate and ECT-induced fever in patients with MMD, aligning with previous findings (33). For instance, Jo et al. (33) reported no considerable difference in the rate of etomidate use between ECT sessions with fever and control sessions without fever (27.8% vs. 21.5%). However, some studies have found a significant association between etomidate and ECT-induced fever in patients with MMD (32, 34, 38). For example, Wang et al. (32) reported that 23.0% of patients receiving etomidate as an anesthetic experienced ECT-induced fever, significantly higher than the 0% incidence in those not administered etomidate. Moreover, Li et al. (38) found that the prevalence of ECT-induced fever was significantly greater among patients receiving etomidate compared to those receiving propofol (46.7% vs. 16.7%). The discrepancies between this study and previous studies (32, 38) have been partly attributed to differences in methodology, such as the definition of fever and the dose of etomidate. For example, the dose of etomidate was administered at 0.3 mg/kg in Wang et al.’s study (32) and 0.2–0.3 mg/kg in Li et al.’s study (38). Therefore, the current evidence does not conclusively determine whether etomidate is significantly associated with ECT-induced fever in patients with MMD. As of September 2023, etomidate was classified as a Class II psychotropic drug by the National Medical Products Administration, the Ministry of Public Security, and the National Health Commission in China (59). This classification led to restricted clinical use of etomidate. Exploring alternative anesthetic agents for ECT is essential. Esketamine or ketamine has been identified as an effective and safe anesthetic for the induction of general anesthesia during ECT, with established antidepressant properties (60–62). However, the association between adjunctive esketamine or ketamine anesthesia in ECT and the incidence of ECT-induced fever remains unexamined.

This study has several limitations worth noting. First, the small sample size restricts the ability to detect significant differences between the case and control groups. Second, unlike previous research (33), this study did not compare the risk factors between ECT sessions with fever and those without, nor were laboratory test results collected. Third, this study was conducted at a single center, which may limit the generalizability of these findings. It was necessary to conduct multicenter studies with a larger and more diverse patient population. Fourth, the prevalence of ECT-induced fever and its risk factors for specific diagnoses such as schizophrenia, BD, or MDD have not been analyzed. Fifth, several key factors (e.g., EEG seizure duration, current intensity, and stimulation duration) that might be linked to ECT-induced fever were neither recorded nor analyzed in this study. Sixth, the control group in this study was chosen based solely on age matching and the absence of post-ECT fever, without considering other factors like comorbidities or medication.

## 5 Conclusions

The findings of this study indicate that the prevalence of ECT-induced fever is relatively low. Moreover, paliperidone and quetiapine were identified as significant independent factors associated with ECT-induced fever in patients with MMD. However, etomidate did not emerge as an essential predictor of ECT-induced fever in this population.

## Data Availability

The raw data supporting the conclusions of this article will be made available by the corresponding author upon reasonable request.

## References

[1] Sun X Ge J Meng H Chen Z Liu D. The influence of social support and care burden on depression among caregivers of patients with severe mental illness in rural areas of sichuan, China. Int J Environ Res Public Health. (2019) 16:1961. doi: 10.3390/ijerph16111961 31159499 PMC6603995

[2] Zhang L Cao XL Wang SB Zheng W Ungvari GS Ng CH. The prevalence of bipolar disorder in China: a meta-analysis. J Affect Disord. (2017) 207:413–21. doi: 10.1016/j.jad.2016.08.062 27771597

[3] Zhong BL Ruan YF Xu YM Chen WC Liu LF. Prevalence and recognition of depressive disorders among chinese older adults receiving primary care: a multi-center cross-sectional study. J Affect Disord. (2020) 260:26–31. doi: 10.1016/j.jad.2019.09.011 31493635

[4] Charlson FJ Ferrari AJ Santomauro DF Diminic S Stockings E Scott JG. Global epidemiology and burden of schizophrenia: findings from the global burden of disease study 2016. Schizophr Bull. (2018) 44:1195–203. doi: 10.1093/schbul/sby058 PMC6192504 29762765

[5] Vos T Abajobir AA AAbbafati C AAbbas KM AAbate KH Abd-Allah F. Global, regional, and national incidence, prevalence, and years lived with disability for 328 diseases and injuries for 195 countries, 1990-2016: a systematic analysis for the global burden of disease study 2016. Lancet (London England). (2017) 390:1211–59. doi: 10.1016/s0140-6736(17)32154-2 PMC5605509 28919117

[6] Morris K Nami M Bolanos JF Lobo MA Sadri-Naini M Fiallos J. Neuroscience20 (brain20, spine20, and mental20) health initiative: a global consortium addressing the human and economic burden of brain, spine, and mental disorders through neurotech innovations and policies. J Alzheimer’s Disease: JAD. (2021) 83:1563–601. doi: 10.3233/jad-215190 34487051

[7] Hock RS Or F Kolappa K Burkey MD Surkan PJ Eaton WW. A new resolution for global mental health. Lancet (London England). (2012) 379:1367–8. doi: 10.1016/s0140-6736(12)60243-8 PMC4767178 22500865

[8] Zhong BL Xu YM Xie WX Li Y. Can p300 aid in the differential diagnosis of unipolar disorder versus bipolar disorder depression? a meta-analysis of comparative studies. J Affect Disord. (2019) 245:219–27. doi: 10.1016/j.jad.2018.11.010 30412774

[9] Xu YM Li F Liu XB Zhong BL. Depressive symptoms in chinese male inpatients with schizophrenia: prevalence and clinical correlates. Psychiatry Res. (2018) 264:380–4. doi: 10.1016/j.psychres.2018.04.016 29677621

[10] Zhang XY Chen HD Liang WN Yang XH Cai DB Huang X. Adjunctive magnetic seizure therapy for schizophrenia: a systematic review. Front Psychiatry. (2021) 12:813590. doi: 10.3389/fpsyt.2021.813590 35082705 PMC8785398

[11] Gogulski J Ross JM Talbot A Cline CC Donati FL Munot S. Personalized repetitive transcranial magnetic stimulation for depression. Biol Psychiatry Cogn Neurosci Neuroimaging. (2023) 8:351–60. doi: 10.1016/j.bpsc.2022.10.006 36792455

[12] Yi S Wang Q Wang W Hong C Ren Z. Efficacy of repetitive transcranial magnetic stimulation (rtms) on negative symptoms and cognitive functioning in schizophrenia: an umbrella review of systematic reviews and meta-analyses. Psychiatry Res. (2024) 333:115728. doi: 10.1016/j.psychres.2024.115728 38232567

[13] Herrera-Melendez AL Bajbouj M Aust S. Application of transcranial direct current stimulation in psychiatry. Neuropsychobiology. (2020) 79:372–83. doi: 10.1159/000501227 31340213

[14] Valiengo L Goerigk S Gordon PC Padberg F Serpa MH Koebe S. Efficacy and safety of transcranial direct current stimulation for treating negative symptoms in schizophrenia: a randomized clinical trial. JAMA Psychiatry. (2020) 77:121–9. doi: 10.1001/jamapsychiatry.2019.3199 PMC6802484 31617873

[15] Wu H Jiang J Cao X Wang J Li C. Magnetic seizure therapy for people with schizophrenia. Cochrane Database Syst Rev. (2023) 6:Cd012697. doi: 10.1002/14651858.CD012697.pub2 37272857 PMC10241155

[16] Mosolov S Born C Grunze H. Electroconvulsive therapy (ect) in bipolar disorder patients with ultra-rapid cycling and unstable mixed states. Medicina (Kaunas Lithuania). (2021) 57:624. doi: 10.3390/medicina57060624 34203943 PMC8232811

[17] Dong M Zhu XM Zheng W Li XH Ng CH Ungvari GS. Electroconvulsive therapy for older adult patients with major depressive disorder: a systematic review of randomized controlled trials. Psychogeriatrics: Off J Japanese Psychogeriatric Society. (2018) 18:468–75. doi: 10.1111/psyg.12359 30073725

[18] Huang XB Zheng W. Ketamine and electroconvulsive therapy for treatment-refractory depression. Alpha Psychiatry. (2023) 24:244–6. doi: 10.5152/alphapsychiatry.2023.231358 PMC10837519 38313444

[19] Singla H Grover S. Electroconvulsive therapy in an elderly patient with severe aortic stenosis: a case report and review of literature. Indian J psychol Med. (2018) 40:288–91. doi: 10.4103/ijpsym.Ijpsym_152_17 PMC5968655 29875541

[20] Jeong SH Youn T Lee Y Jang JH Jeong YW Kim YS. Initial seizure threshold in brief-pulse bilateral electroconvulsive therapy in patients with schizophrenia or schizoaffective disorder. Psychiatry Invest. (2019) 16:704–12. doi: 10.30773/pi.2019.06.20.2 PMC6761792 31429220

[21] Tang YL Jiang W Ren YP Ma X Cotes RO McDonald WM. Electroconvulsive therapy in China: clinical practice and research on efficacy. J ECT. (2012) 28:206–12. doi: 10.1097/YCT.0b013e31825957b1 22801297

[22] Gutowski B Bomasang-Layno E. The role of acetylcholinesterase inhibitors in the treatment of prolonged postelectroconvulsive therapy delirium. Case Rep Psychiatry. (2022) 2022:6966882. doi: 10.1155/2022/6966882 35677728 PMC9170446

[23] İlhan Atagün M Atay Canbek Ö. A systematic review of the literature regarding the relationship between oxidative stress and electroconvulsive therapy. Alpha Psychiatry. (2022) 23:47–56. doi: 10.5152/alphapsychiatry.2021.21584 36426296 PMC9597066

[24] Rönnqvist I Nilsson FK Nordenskjöld A. Electroconvulsive therapy and the risk of suicide in hospitalized patients with major depressive disorder. JAMA Netw Open. (2021) 4:e2116589. doi: 10.1001/jamanetworkopen.2021.16589 34287633 PMC8295734

[25] Lin HT Liu SK Hsieh MH Chien YL Chen IM Liao SC. Impacts of electroconvulsive therapy on 1-year outcomes in patients with schizophrenia: a controlled, population-based mirror-image study. Schizophr Bull. (2018) 44:798–806. doi: 10.1093/schbul/sbx136 29036711 PMC6007329

[26] Slade EP Jahn DR Regenold WT Case BG. Association of electroconvulsive therapy with psychiatric readmissions in us hospitals. JAMA Psychiatry. (2017) 74:798–804. doi: 10.1001/jamapsychiatry.2017.1378 28658489 PMC5710550

[27] Ying YB Jia LN Wang ZY Jiang W Zhang J Wang H. Electroconvulsive therapy is associated with lower readmission rates in patients with schizophrenia. Brain Stimul. (2021) 14:913–21. doi: 10.1016/j.brs.2021.05.010 34044182

[28] Deng CJ Nie S Mai JX Huang X Huang XB Zheng W. Electroconvulsive therapy knowledge and attitudes among patients and caregivers in south China: a preliminary study. Front Psychiatry. (2023) 14:1145301. doi: 10.3389/fpsyt.2023.1145301 36993925 PMC10040676

[29] Zong QQ Qi H Wang YY Zhang C Balbuena L Ungvari GS. Knowledge and attitudes of adolescents with psychiatric disorders and their caregivers towards electroconvulsive therapy in China. Asian J Psychiatry. (2020) 49:101968. doi: 10.1016/j.ajp.2020.101968 32135482

[30] Kumar S Mulsant BH Liu AY Blumberger DM Daskalakis ZJ Rajji TK. Systematic review of cognitive effects of electroconvulsive therapy in late-life depression. Am J Geriatr Psychiatry: Off J Am Assoc Geriatr Psychiatry. (2016) 24:547–65. doi: 10.1016/j.jagp.2016.02.053 27067067

[31] Holtzheimer PE 3rd Nemeroff CB. Emerging treatments for depression. Expert Opin Pharmacother. (2006) 7:2323–39. doi: 10.1517/14656566.7.17.2323 17109609

[32] Wang X Jiang H Shen S Jia Y. Effect of propofol for prevention of side effects in patients after mect (in chinese). J Psychiatry. (2015) 28:334–5. doi: 10.3969/j.issn.2095-9346.2015.05.004

[33] Jo YT Lee J Joo YH. Fever as a side effect after electroconvulsive therapy. Neuropsychobiology. (2022) 81:19–27. doi: 10.1159/000511542 34233323

[34] Bryson EO Pasculli RM Briggs MC Popeo D Aloysi AS Kellner CH. Febrile reaction with elevated cpk after a single electroconvulsive therapy (ect) in an adolescent patient with severe bipolar disorder. J ECT. (2012) 28:70–1. doi: 10.1097/YCT.0b013e31823dfeb0 22343589

[35] Cheung EFC. Benign recurrent febrile reactions induced by electroconvulsive therapy in an adolescent chinese with catatonic schizophrenia:a case report. Acta Psychopathol. (2015) 1:2. doi: 10.4172/2469-6676.100002

[36] Xiao A Liang Q Shuai S Chen M. Observations on the adverse effects of modified electroconvulsive therapy in psychiatric patients (in chinese). J Nurs Sci. (2001) 16(8):485–6.

[37] Xie Q Ye B Chen H Wu W. An analysis of fever in patients after modified electroconvulsive therapy (in chinese). J Jinggangshan Univ (Science Technology). (2009) 30:105–6.

[38] Li S Deng P Li Y Luo W Zhang Q. Comparison of fever after conventional and modified electroconvulsive therapy (in chinese). Military Med J South China. (2014) 28:283–4. doi: 10.3969/j.issn.1009-2595.2014.03.031

[39] WHO. Guidelines for the treatment of malaria. Switzerland: WHO (2006). Available at: www.who.int (Accessed January 01, 2006).

[40] Ogoina D. Fever, fever patterns and diseases called ‘fever’–a review. J Infect Public Health. (2011) 4:108–24. doi: 10.1016/j.jiph.2011.05.002 21843857

[41] Chen YC Kuo YC Chen MC Zhang YD Chen CL Le PH. Case-control study of clostridium innocuum infection, Taiwan. Emerg Infect Dis. (2022) 28:599–607. doi: 10.3201/eid2803.204421 35195517 PMC8888209

[42] Zheng W Jiang ML He HB Li RP Li QL Zhang CP. A preliminary study of adjunctive nonconvulsive electrotherapy for treatment-refractory depression. Psychiatr Quarterly. (2021) 92:311–20. doi: 10.1007/s11126-020-09798-3 32661940

[43] Petrides G Fink M. The “half-age” stimulation strategy for ect dosing. Convulsive Ther. (1996) 12:138–46. 8872401

[44] Virit O Ayar D Savas HA Yumru M Selek S. Patients’ and their relatives’ attitudes toward electroconvulsive therapy in bipolar disorder. J ECT. (2007) 23:255–9. doi: 10.1097/yct.0b013e318156b77f 18090699

[45] Gomez J. Subjective side-effects of ect. Br J Psychiatry: J Ment Sci. (1975) 127:609–11. doi: 10.1192/bjp.127.6.609 1201457

[46] Li Y An FR Zhu H Chiu HF Ungvari GS Ng CH. Knowledge and attitudes of patients and their relatives toward electroconvulsive therapy in China. Perspect Psychiatr Care. (2016) 52:248–53. doi: 10.1111/ppc.12124 26033408

[47] Lyng JW Guyette FX Levy M Bosson N. Prehospital manual ventilation: an naemsp position statement and resource document. Prehospital Emergency Care. (2022) 26:23–31. doi: 10.1080/10903127.2021.1981506 35001826

[48] Le H Yue J Wen S. One case report of fever after convulsive electroconvulsive therapy (in chinese). Chin Med Care Repos. (2022) 04:E02568–E.

[49] Deng CJ Nie S Mai JX Zou DC Deng W Huang X. Narrative review and consensus recommendations for the use of transnasal humidified rapid-insufflation ventilatory exchange in modified electroconvulsive therapy. Alpha Psychiatry. (2024) 25:282–9. doi: 10.5152/alphapsychiatry.2024.231463 PMC11117428 38798804

[50] Riva T Meyer J Theiler L Obrist D Bütikofer L Greif R. Measurement of airway pressure during high-flow nasal therapy in apnoeic oxygenation: a randomised controlled crossover trial. Anaesthesia. (2021) 76:27–35. doi: 10.1111/anae.15224 32776518

[51] Jonker Y Rutten DJ van Exel ER Stek ML de Bruin PE Huitink JM. Transnasal humidified rapid-insufflation ventilatory exchange during electroconvulsive therapy: a feasibility study. J ECT. (2019) 35:110–4. doi: 10.1097/yct.0000000000000556 30461537

[52] Voronova IP. 5-ht receptors and temperature homeostasis. Biomolecules. (2021) 11:1914. doi: 10.3390/biom11121914 34944557 PMC8699715

[53] Dev V Raniwalla J. Quetiapine: a review of its safety in the management of schizophrenia. Drug Safety. (2000) 23:295–307. doi: 10.2165/00002018-200023040-00003 11051217

[54] Nothdurfter C Schmotz C Sarubin N Baghai TC Laenger A Lieb M. Effects of escitalopram/quetiapine combination therapy versus escitalopram monotherapy on hypothalamic-pituitary-adrenal-axis activity in relation to antidepressant effectiveness. J Psychiatr Res. (2014) 52:15–20. doi: 10.1016/j.jpsychires.2014.01.013 24513501

[55] Alp A Doğan MK Eroğlu E Yildiz M Gürel ŞC Özer S. Transient fever response after ect in a patient with catatonic schizophrenia: a case report. Turk psikiyatri dergisi = Turkish J Psychiatry. (2024) 35:78–82. doi: 10.5080/u26972 PMC11003368 38556940

[56] Minwalla HD Wrzesinski P Desforges A Caskey J Wagner B Ingraffia P. Paliperidone to treat psychotic disorders. Neurol Int. (2021) 13:343–58. doi: 10.3390/neurolint13030035 PMC8396046 34449689

[57] Zheng X Hasegawa H. Central dopaminergic neurotransmission plays an important role in thermoregulation and performance during endurance exercise. Eur J Sport Sci. (2016) 16:818–28. doi: 10.1080/17461391.2015.1111938 26581447

[58] MacDowell KS Munarriz-Cuezva E Caso JR Madrigal JL Zabala A Meana JJ. Paliperidone reverts toll-like receptor 3 signaling pathway activation and cognitive deficits in a maternal immune activation mouse model of schizophrenia. Neuropharmacology. (2017) 116:196–207. doi: 10.1016/j.neuropharm.2016.12.025 28039001

[59] Administration NMP China TMoPSotPsRo China NHCotPsRo. National medical products administration and the national health commission notice on strengthening the management of etomidate and modafinil drugs. China: National Medical Products Administration (2023). Available at: https://www.nmpa.gov.cn/xxgk/fgwj/gzwj/gzwjyp/20231007154014186.html (Accessed September 28, 2023).

[60] Zeng QB Zou DC Huang XB Shang DW Huang X Yang XH. Efficacy and safety of esketamine versus propofol in electroconvulsive therapy for treatment-resistant depression: a randomized, double-blind, controlled, non-inferiority trial. J Affect Disord. (2024) 368:320–8. doi: 10.1016/j.jad.2024.09.038 39265871

[61] Zang X Zhang J Hu J Mo X Zheng T Ji J. Electroconvulsive therapy combined with esketamine improved depression through pi3k/akt/glt-1 pathway. J Affect Disord. (2024) 368:282–94. doi: 10.1016/j.jad.2024.08.123 39265873

[62] Ren L Chen Q Gao J Liu Y Tao Y Li X. Clinical efficacy of adjunctive esketamine anesthesia in electroconvulsive therapy for major depressive disorders: a pragmatic, randomized, controlled trial. Psychiatry Res. (2024) 335:115843. doi: 10.1016/j.psychres.2024.115843 38461645

